# Screen or not to screen for peripheral arterial disease: guidance from a decision model

**DOI:** 10.1186/1471-2458-14-89

**Published:** 2014-01-29

**Authors:** Anil Vaidya, Manuela A Joore, Arina J ten Cate-Hoek, Hugo ten Cate, Johan L Severens

**Affiliations:** 1 Department of Clinical Epidemiology and Medical Technology Assessment (KEMTA), Maastricht University Medical Centre, Maastricht, The Netherlands; 2 School for Public Health and Primary Care (CAPHRI), Maastricht University, Maastricht, The Netherlands; 3 Laboratory for Clinical Thrombosis and Haemostasis, Department of Internal Medicine, Cardiovascular Research Institute Maastricht (CARIM), Maastricht University Medical Centre, Maastricht, the Netherlands; 4 Institute of Health Policy & Management, Erasmus University Rotterdam, Rotterdam, the Netherlands

**Keywords:** Cost-effectiveness, Peripheral arterial disease, Ankle brachial index, Decision model

## Abstract

**Background:**

Asymptomatic Peripheral Arterial Disease (PAD) is associated with greater risk of acute cardiovascular events. This study aims to determine the cost-effectiveness of one time only PAD screening using Ankle Brachial Index (ABI) test and subsequent anti platelet preventive treatment (low dose aspirin or clopidogrel) in individuals at high risk for acute cardiovascular events compared to no screening and no treatment using decision analytic modelling.

**Methods:**

A probabilistic Markov model was developed to evaluate the life time cost-effectiveness of the strategy of selective PAD screening and consequent preventive treatment compared to no screening and no preventive treatment. The analysis was conducted from the Dutch societal perspective and to address decision uncertainty, probabilistic sensitivity analysis was performed. Results were based on average values of 1000 Monte Carlo simulations and using discount rates of 1.5% and 4% for effects and costs respectively. One way sensitivity analyses were performed to identify the two most influential model parameters affecting model outputs. Then, a two way sensitivity analysis was conducted for combinations of values tested for these two most influential parameters.

**Results:**

For the PAD screening strategy, life years and quality adjusted life years gained were 21.79 and 15.66 respectively at a lifetime cost of 26,548 Euros. Compared to no screening and treatment (20.69 life years, 15.58 Quality Adjusted Life Years, 28,052 Euros), these results indicate that PAD screening and treatment is a dominant strategy. The cost effectiveness acceptability curves show 88% probability of PAD screening being cost effective at the Willingness To Pay (WTP) threshold of 40000 Euros. In a scenario analysis using clopidogrel as an alternative anti-platelet drug, PAD screening strategy remained dominant.

**Conclusion:**

This decision analysis suggests that targeted ABI screening and consequent secondary prevention of cardiovascular events using low dose aspirin or clopidogrel in the identified patients is a cost-effective strategy. Implementation of targeted PAD screening and subsequent treatment in primary care practices and in public health programs is likely to improve the societal health and to save health care costs by reducing catastrophic cardiovascular events.

## Background

Peripheral Arterial Disease (PAD) is a common disorder with a prevalence estimated at 16% in those aged over 55 years and 29% in high-risk groups [1,2]. PAD is a sign of widespread atherosclerosis also affecting coronary, cerebral and renal arteries. PAD is associated with a significant reduction in Quality of Life (QoL) and greater risk of acute cardiovascular events [3,4]. The increased risk for cardiovascular morbidity, such as myocardial infarction and stroke, and increased risk for mortality is also observed in asymptomatic patients [5]. The Cardiovascular consequences of PAD, are known to be expensive and contribute substantially to national health care costs [6].

European Society of Cardiology (ESC), American Heart Association (AHA) and American College of Cardiology (ACC) clinical practice guidelines recommend low dose aspirin to reduce the cardiovascular events and mortality in symptomatic PAD patients [7,8]. Clopidogrel is recommended as an effective alternative anti-platelet therapy to aspirin for secondary prevention in PAD [8]. Ankle Brachial Index (ABI) is used for detection of PAD. The ABI is calculated by measuring both arm and leg blood pressure (at ankle level). This reliable and inexpensive test is highly sensitive and specific for PAD. However, ABI screening in asymptomatic patients is a controversial topic among the health professionals. United States preventive services task force (USPSTF) assigned a “D” recommendation to the routine screening of PAD [9]. This recommendation is intensely debated and a routine targeted screening for PAD is recommended to increase the frequency of diagnosis, improve the use of recommended medical therapies, and consequently reduce cardiovascular morbidity and mortality rates [10]. Researchers have voiced that ‘it’s not just about legs’ and ABI measurement in asymptomatic individuals should be regarded as the biomarker of cardiovascular disease risk [11]. While expansion of the evidence base for PAD screening is recommended in the year 2011 focussed update of the guidelines [12], targeted ABI screening is recommended by all professional vascular societies including the ACC [8].

The Rotterdam study has identified risk factors that are most strongly associated with PAD such as older age, cigarette smoking, diabetes mellitus, hypercholesterolemia and hypertension [13]. These risk factors can be used to guide targeted ABI screening in a general population over 55 years of age.

In current health care practice, asymptomatic PAD often remains undiagnosed and opportunities for secondary prevention are missed [2]. Therefore, there is a clinical need of early detection of asymptomatic PAD and for the initiation the appropriate preventive treatment in a high risk population. Although, prevention and subsequent treatment comes at a certain cost the secondary prevention of cardio-vascular consequences in PAD patients may at the same time improve prognosis and save healthcare resources. This study aims to determine the cost-effectiveness of PAD screening using ABI and subsequent preventive treatment in high risk individuals at high risk for acute cardiovascular events with low dose aspirin or clopidogrel compared to no screening and treatment.

## Methods

A model-based economic evaluation of targeted ABI screening in high-risk group was performed taking lifetime costs and health effects in account for a Dutch health care setting. Microsoft Excel 2010^©^ software was used for this modelling work.

Future costs and outcomes were discounted at the rates of 4% and 1.5% respectively, as per the Dutch guidelines for pharmaco-economic research [14]. This study was conducted from the societal perspective and indirect costs (productivity loss) were taken into account.

### Model approach

The hypothetical population consists of asymptomatic males and females aged 55 years with at least one of the vascular risk factors identified in the Rotterdam study [13].

The intervention is one time screening in a high risk population at the age of 55 years using ABI, the current standard test to detect PAD in primary care. Screening in principle is intended to take place in the general practitioner’s office, in a similar manner as ‘The prevention visit’ for the cardiovascular risk assessment, defined in the Dutch College of General Practitioners' practice guideline [15]. We modelled that all ABI test positive patients will receive preventive treatment with low dose aspirin in the base case analysis. In a scenario analysis low dose aspirin is replaced with clopidogrel as preventive treatment in patients with a positive test.

ABI screening is compared to no screening of the high risk population and preventive treatment is only given to the incidentally diagnosed or incidentally symptomatic patients. Model Outcomes were life years (LYs), quality adjusted life years (QALYs) and costs. The model has a time horizon of a life time as the hypothetical patient cohort was followed until death. The model cycle duration was one year.

### Model structure

**Figure 1:**
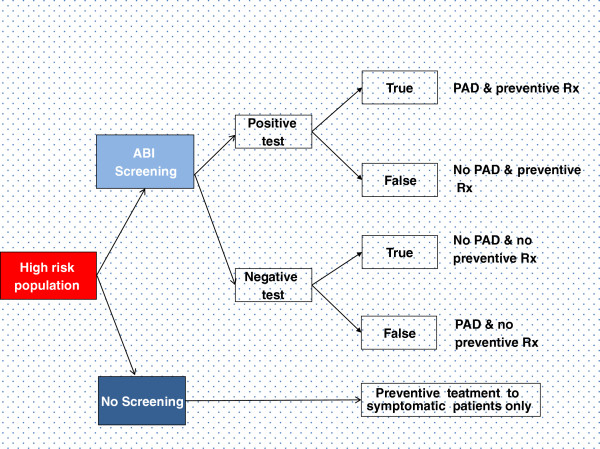
Decision tree.

**Figure 2:**
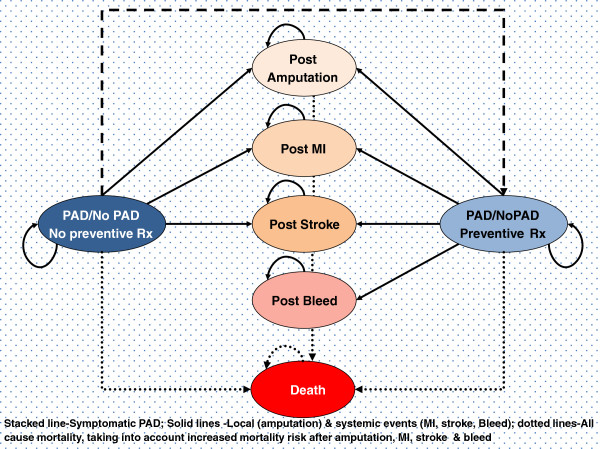
Markov model.

Based on a systematic review of modelling approaches for PAD, we used a combination of two modelling approaches: a decision tree and a Markov state transition model shown in the Figure 1 and Figure 2[16]. The decision tree was used to determine the number of screened individuals falling into the categories of test positive or test negative on the basis of test accuracy and prevalence of PAD. A Markov model was subsequently used to model the on-going risk of cardiovascular events over a lifetime.

The model assumes that the patient is always in one of a finite number of states of health referred to as Markov states. The time horizon of the analysis is divided into equal increments of time, referred to as Markov cycles, in this case one year. During each cycle, the cohort of patients is redistributed over the Markov states, thus theoretically a patient may make a transition from one state to another. Each state is assigned a utility and a cost. Total costs and utility for screening versus no-screening are calculated depending upon the distribution of the cohort over the Markov states and the length of time spent in each state [17].

We included all the relevant cardiovascular health states in the Markov model (no PAD, asymptomatic PAD, and symptomatic PAD, post amputation, post myocardial infarction, post stroke, post bleed-in treated patients and the absorbing state of death). Risk reductions of cardiovascular events and mortality as well as increased bleeding risk caused by the preventive anti-platelet treatment were modelled accordingly in our model. All the model parameters are shown in Table 1.

**Table 1:** Model parameters and distribution used in the probabilistic sensitivity analyses

**Model parameter**	**Value**	**Probability distribution**	**Moments of the probability distribution ά/min, β/max**	**Source**
**Discount rates**				
Cost discount rate	4%	Fixed	-	[14]
Outcome discount rate	1.5%	Fixed	-	[14]
Costs (Euros)*				
Cost of ankle brachial index test	74†	BETA Pert	55.7;92.8	MUMC‡
Annual cost of PAD treatment	2369	GAMMA	325.09;7.29	[18]
Annual cost of Aspirin	10	Fixed		[19]
Annual cost of Clopidogrel	19	Fixed		[19]
Costs of Amputation	14343†	BETA Pert	10683;17804	[20]
Cost of AMI in first year	25328	GAMMA	100;253.27	[21]
Annual costs of MI treatment in subsequent years	3584	GAMMA	99.92;35.86	[21]
Cost of stroke in first year	27964	GAMMA	99.99;279.66	[21]
Annual costs of treatment of stroke in subsequent years	10646	GAMMA	99.99;106.47	[21]
Costs of bleeding	3457	GAMMA	99.87;34.61	[21]
**ABI test accuracy**				
Sensitivity	0.90†	BETA Pert	0.68;1	[22]
Specificity	0.95†	BETA Pert	0.71;1	[22]
**Incidence/prevalence of PAD**				
Prevalence of PAD	0.184	BETA	1372;6082	[6]
Annual incidence of PAD in 55–64 years aged	0.005	BETA	See Additional file 1	[23]
Annual incidence of PAD in 65–74 years aged	0.007	BETA	See Additional file 1	[23]
Annual incidence of PAD in 75–84 years aged	0.008	BETA	See Additional file 1	[23]
Annual incidence of PAD in >85 years aged	0.010	BETA	See Additional file 1	[23]
**Event probabilities**				
Probability of amputation in patients with no PAD	0.003	BETA	32;11734	[24]
Probability of AMI in patients with no PAD	0.008	BETA	89;11677	[24]
Probability of stroke in patients with no PAD	0.008	BETA	94;11672	[24]
Probability of amputation in PAD patients	0.016	BETA	140;8441	[24]
Probability of AMI in PAD patients	0.013	BETA	111;8470	[24]
Probability of stroke in PAD patients	0.019	BETA	165;8416	[24]
Probability of symptomatic PAD	0.3	BETA	138;320	[25]
Relative risk in PAD patients on low dose aspirin	0.78	BETA	25.45;7.2	[26]
Probability of bleeding in PAD patients on aspirin	0.026	BETA	255;9311	[27]
Relative risk in PAD patients on Clopidogrel	0.616	BETA	See Additional file 1	[28]
Probability of bleeding in PAD patients on Clopidogrel	0.020	BETA	191;9386	[27]
**Mortality in ‘untreated’ patients**				
Annual probability of death in PAD patients	0.037	BETA	323;8258	[24]
Probability of death in post Amputation	0.155	BETA	4297;21281	[29]
annual probability of death in post MI alive patients	0.028	BETA	521;17492	[24]
annual probability of death in post stroke alive patients	0.031	BETA	1212;37390	[24]
**Utility**				
PAD	0.652	BETA	0.8;0.4	[30]
Amputation	0.45	BETA	210.8;257.7	[31]
Post MI	0.671	BETA	69.3;34	[30]
Post stroke	0.519	BETA	2.7;2.5	[30]
Post bleed	0.627	BETA	405.6;241.13	[30]

*All costs were converted to 2012 Dutch costs using harmonized index of consumer prices (HICP).

‡This cost was obtained from the Financial department of Maastricht University Medical Centre.

†Mode for BETA Pert distribution.

### Transition probabilities

Transition probabilities for PAD patients were calculated from the REACH (Reduction of Athero-thrombosis for Continued Health) registry. This multinational database contains 68,375 consecutive outpatients from 5587 physician practices in 44 countries and was enrolled between December 2003 and June 2004 [24]. Patients on anti-platelet preventive treatment have reduced cardiovascular morbidity and mortality but on the other hand this treatment increases the risk of bleeding in the recipients. Transition probabilities for cardiovascular events in patients receiving Aspirin were calculated from a meta-analysis of randomised trials ‘Aspirin for the Prevention of Cardiovascular Events in Patients With Peripheral Artery Disease’ [26]. Probabilities for Clopidogrel were calculated from a Cochrane review of anti-platelet agents for intermittent claudication [28]. Bleeding risks in patients receiving low dose aspirin or clopidogrel were assigned from a randomized, blinded, trial of clopidogrel versus aspirin in patients at risk of ischemic events (CAPRIE) [27].

### Costs

The acute phase costs and subsequent costs of cardiovascular events were taken from Thurston et al. [21] and the costs of amputation and of cardiovascular death are from Oostenbrink et al. [20]. The annual costs for an average PAD patient were published by van Asselt et al. [18] Dutch costs of anti-platelet medications aspirin and clopidogrel were obtained from the medicine cost website in the Netherlands [19]. Travel costs for attending the PAD screening were calculated based on the average distance to a primary practice. The average distance to a Dutch primary practice is 1.1 KM [32]. Cost of a session at a primary care physician and productivity loss for a 55 years old individual in the Netherlands are published in the Dutch manual for costing in economic evaluations [33]. All costs used in the model were converted to Year 2012 Dutch costs using harmonized index of consumer prices data from the Dutch bureau of statistics [34].

### Utilities

Since Dutch utility scores for the health states defined in our model were not found in the literature, we used Sullivan et al. to estimate utilities for all the health states except amputation [30]. The utility of an amputee using standard gamble method was taken from Berry et al. [31].

### Analysis

Discounted and undiscounted expected life years and QALYs (1.5% discount rate), and costs (discount rate 4%) for each strategy were calculated. Based on the discounted expected values, the Incremental Cost Effectiveness Ratios (ICERs) of the screening and treatment strategy were calculated over the standard existing practice of no screening and preventive treatment with low dose aspirin for incidentally diagnosed or symptomatic patients only.

The results of cost-effectiveness analysis were based on Probabilistic Sensitivity Analysis (PSA). Results of 1000 Monte Carlo simulations were graphically displayed in the form of cost-effectiveness planes (CE planes) and the subsequent probability of being cost-effective at different values of willingness to pay (WTP) thresholds was shown as cost-effectiveness acceptability curves (CEACs).

One way sensitivity analyses were performed to identify the two most influential model parameters affecting model outputs. For this purpose, upper and lower limits of 95% confidence interval of model parameters were used. Then, a two way sensitivity analysis was conducted for combinations of values tested for these two most influential parameters.

### Scenario analysis

We performed a scenario analysis by replacing routinely prescribed low dose aspirin with a relatively new anti-platelet drug clopidogrel for the secondary prevention of cardiovascular events in identified PAD patients.

## Results

### Cost-effectiveness analysis

**Figure 3:**
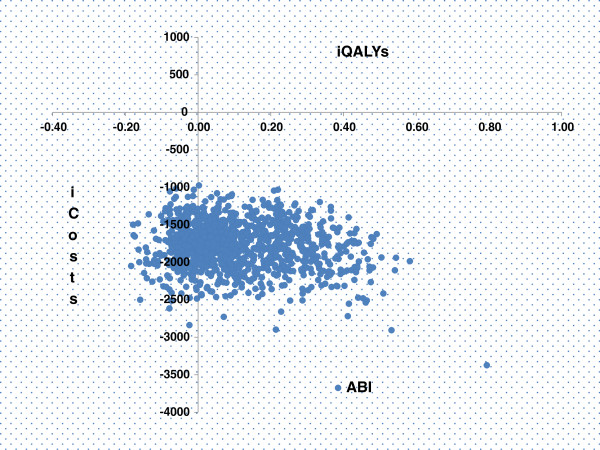
**incremental Cost-effectiveness planes.** Shows that PAD screening followed by low dose aspirin treatment was a dominant strategy (less costly, more effective) in 88% of simulations.

**Figure 4:**
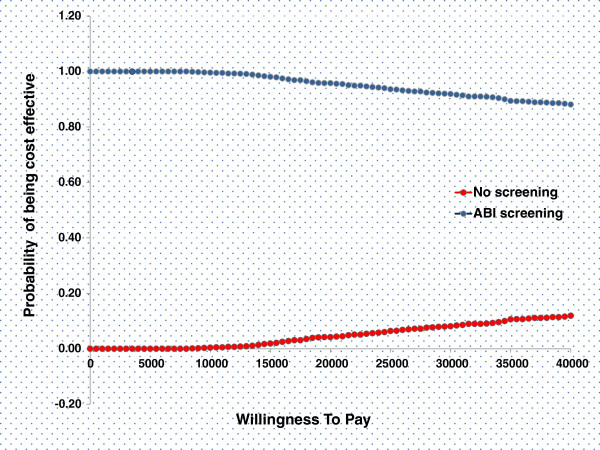
**Cost-effectiveness acceptability curves.** The acceptability curves show that ABI Screening followed by treatment with low dose aspirin remains 100% cost-effective at willingness to pay (WTP) thresholds of zero to 11000 Euros and 88% cost-effective at a WTP threshold of 40000 Euros.

The expected model outcomes show that the targeted ABI screening and treatment with low dose aspirin produce 21.79 mean LYs and 15.66 mean QALYs for a cost of 26,548 Euros. The cost of PAD screening and treatment followed by low dose aspirin was 1503 Euros lower compared to ‘no screening’ and 0.07 QALYs were gained (Table 2). Therefore, ABI screening followed by preventive treatment with low dose aspirin is a dominant strategy. The relationship between costs and effects and the uncertainty surrounding these estimates are shown in the cost effectiveness planes in the Figure 3. Monte Carlo simulation shows that the 88% of ICER dots are in the right lower quadrant indicating that the strategy ‘PAD screening’ tended to have favourable health outcomes against lower costs in comparison with a strategy of ‘no screening’. The probability of being cost effective at different values of willingness to pay (WTP) thresholds was shown as cost effectiveness acceptability curves (CEACs) in the Figure 4. The curves show the probability of PAD screening being cost effective at a range of Willingness To Pay thresholds. There is 88% probability of PAD screening being cost effective at the WTP of 40000 Euros.

**Table 2:** Results – base case analysis and scenario analysis

**PROBABILISTIC RESULTS (discounted)**								
**Diagnostic test**	**Treatment**	**Costs**	**LYs**	**QALYs**	**iCosts**	**iLY**	**iQALYs**	**iCERs**
**Base case**								
No screen	Low dose aspirin	28052	20.69	15.58				
ABI screening	Low dose aspirin	26548	21.79	15.66	-1503	1.10	0.007	Dominant
**Scenario analysis with Clopidogrel**								
No screen	Clopidogrel	29464	22.33	15.95				
ABI	Clopidogrel	27681	22.57	16.17	-1783	0.24	0.22	Dominant
**PROBABILISTIC RESULTS (undiscounted)**								
**Diagnostic test**	**Treatment**	**Costs**	**LYs**	**QALYs**	**iCosts**	**iLY**	**iQALYs**	
**Base case**								
No screen	Low dose aspirin	63155	26.32	19.40				
ABI screening	Low dose aspirin	59544	27.47	19.50	-3611	1.15	0.11	
**Scenario analysis with Clopidogrel**								
No screen	Clopidogrel	67799	28.30	19.96				
ABI	Clopidogrel	63759	28.66	20.27	-4039	0.36	0.31	

Scenario analysis with the use of clopidogrel as an alternative anti-platelet therapy produced similar results indicating dominance over ‘no screening’ (Table 2).

The one way sensitivity analysis identified PAD prevalence and relative risk reduction by low dose aspirin in the treated PAD patients, as the two most influential model parameters. Although the ICER for ABI screening remained dominant for all the variations in parameter values, two way sensitivity analysis varying PAD prevalence and relative risk reduction by aspirin showed a consistent QALY gain by either increasing the prevalence of PAD or relative risk reduction by low dose aspirin.

## Discussion

Our cost-effectiveness model output suggests that targeted screening of high risk individuals and consequent secondary prevention of cardiovascular events by anti-platelet medication is cost effective and results in significant health gain by reducing cardiovascular events in PAD patients.

The analysis has been performed from societal perspective and all direct and indirect costs are incorporated for all the health states in the model. Our analysis interprets that PAD screening and anti-platelet preventive treatment is a highly cost-effective intervention. Changing the analysis perspective to health care payer’s, would further strengthen this interpretation. This is the case in countries like the United Kingdom where health care is financed by general taxation, a health care provider’s (National Health Services) perspective is used in pharmaco-economic analyses and only direct costs are covered.

A recent meta-analysis concluded that measurement of the ankle brachial index may improve the accuracy of cardiovascular risk prediction beyond the Framingham Risk Score [35]. After adjustment for the Framingham risk score, the ABI provided significant improvement in predicting cardiovascular risk independent of established risk factors in a broad population. There is unequivocal evidence establishing the importance of targeted ABI screening [36,37].

In our model costs and effects were modeled for aspirin and clopidogrel. The CAPRIE trial data show that clopidogrel is more effective than aspirin in reducing cardiovascular events in the subgroup of patients with PAD [27]. However, with branded clopidogrel (Plavix), cost was a major barrier for longer-term use. Plavix (clopidogrel) lost its patent protection from May 2012 and the generic form of clopidogrel is available at a much lower cost. Since branded Plavix may have been cost-prohibitive in certain non-reimbursement settings, there will likely be an increase in compliance with long-term generic clopidogrel therapy. Economic model results for the use of clopidogrel in PAD patients were in line with previous studies establishing the cost-effectiveness of this drug [38,39]. These results were expected as generic clopidogrel costs only few Euros more than aspirin and provides higher risk reduction from C The limitation of our model is that the costs and health outcomes of only antiplatelet treatment are modeled. Depending upon the identified risk factors in the individual patients many additional medication interventions such as statins and tension lowering medications are prescribed for the medical management of PAD. However, our study focusses on antiplatelet treatment using Aspirin or Clopidogrel as this is the most commonly prescribed medication in almost all the patients. In order to model the additional therapies for each subgroup of high risk patients a much more complex model is needed.

ABI has been in wide use at specialized vascular clinics but its application in primary practice is limited. With a high degree of diagnostic accuracy and as much prognostic information, ABI screening is a cost-effective but underused primary care tool to detect PAD. Mohler et al. found that the time to perform ABI, staff constrains and lack of reimbursement are the most important barriers in its use and appropriate measures are required to deal with these barriers [40]. However, this cost-effective analysis clearly indicates that the ABI screening is a highly cost-effective clinical tool to be applied in the primary care.

In the real world scenario, compliance to the ABI screening and to the preventive treatment thereafter, in an apparently healthy population could pose a challenge. However, factors like the noninvasive nature of the ABI testing and routine primary care visits of high risk individuals are likely to contribute to good compliance. Although lower compliance to the screening programme may affect the overall cost effectiveness results, it is unlikely to negate the cost-effectiveness of ABI screening altogether because of the high probability of the screening programme to be cost-effective as shown in the cost-effectiveness plane (Figure 4).

Our results are in line with a previously published modeling study by Sigvant et al. to assess the cost-effectiveness of various therapeutic agents in asymptomatic PAD patients. Aspirin as one of the preventive therapies produced similar health outcomes in 55 year-old patients as our model [41].

In line with ACC/AHA guidelines, this modeling study is only based upon risk reduction by preventive anti-platelet therapy given to the PAD patients. Additional cardiovascular risk reducing treatments such as exercise therapy, lipid lowering statins and blood pressure lowering drugs are also prescribed in PAD. Further research is required to quantify the consolidated effects of diverse preventive options in a heterogenic PAD population.

## Conclusions

This study has assessed the impact of PAD screening using long term clinical and economic outcomes. The results show that targeted ABI screening and consequent secondary prevention of cardiovascular events using low dose aspirin or clopidogrel is a cost-effective strategy. Our study results provide one of the building blocks of evidence expansion for advocating PAD screening and the promotion of its more widespread use to detect and treat PAD patients. Implementation of targeted PAD screening and subsequent treatment in primary care practices and in public health programs is likely to improve the societal health and to save health care costs by reducing catastrophic cardiovascular events.

## Abbreviations

PAD: Peripheral arterial disease; ABI: Ankle brachial index; WTP: Willingness to pay; QoL: Quality of life; ESC: European Society of Cardiology; AHA: American Heart Association; ACC: American College of Cardiology; USPSTF: United States Preventive Services Task Force; EBM: Evidence Based Medicine; REACH: Reduction of athero-thrombosis for continued health; CAPRIE: Clopidogrel versus aspirin in patients at risk of ischemic events; CE planes: Cost effectiveness planes; LY: Life years; QALY: Quality-adjusted life year; ICER: Incremental cost effectiveness ratio; CEAC: Cost-effectiveness acceptability curve

## Competing interests

None of the authors have any competing interests.

## Authors’ contributions

AV and MJ: Conceived the study, assimilated all necessary data for PAD, performed the cost-effectiveness analysis, interpreted the results and drafted the manuscript. AT and HT: Supplied all data required from the academic hospital and helped to draft the manuscript. JS: Gave advice on all statistical analyses performed, instructed on the study methodology and reviewed the manuscript multiple times. All authors read and approved the final manuscript.

## Pre-publication history

The pre-publication history for this paper can be accessed here:

http://www.biomedcentral.com/1471-2458/14/89/prepub

## Supplementary Material

Additional file 1 Calculation of probabilistic moments for PAD Incidence. Click here for file
