# Supplementary material for: Screen or not to screen for peripheral arterial disease: guidance from a decision model
Source: BMC Public Health. 2014 Jan 29;14:89. doi: 10.1186/1471-2458-14-89 (PMC3912926; doi:10.1186/1471-2458-14-89)
Supplement: Additional file 1 — Calculation of probabilistic moments for PAD Incidence. [file 1471-2458-14-89-S1.docx]

**Appendix 1**

**Calculation of probabilistic moments for PAD Incidence**

Source: Meijer et al 2002[^1^](#_ENREF_1)

Age and sex specific incidence (cases per 1000 person years) with 95% Confidence Interval are reported by Meijer et a . We have calculated Standard Error (SE) for each age block and for both sexes. Using the SE alpha and beta moments for beta distribution were calculated.

A weighted average from both sexes was calculated to have a unisex age specific incidence. To simplify the model input parameter sheet we condensed the age blocks to every ten years instead of originally reported Five years.

|  | **Male** |  | 95% Confidence Interval | |  |  |  |  |
| --- | --- | --- | --- | --- | --- | --- | --- | --- |
| Age | Prob value | Incidence | Upper | Lower | SE | Alpha | Beta | PY |
| 55-59 | 0,006 | 0,004 | 0,008 | 0,002 | 0,002 | 6,37 | 1585,24 | 2004 |
| 60-64 | 0,007 | 0,010 | 0,016 | 0,006 | 0,003 | 14,61 | 1460,93 | 1725 |
| 65-69 | 0,009 | 0,009 | 0,015 | 0,005 | 0,003 | 10,97 | 1207,80 | 1446 |
| 70-74 | 0,014 | 0,013 | 0,022 | 0,008 | 0,004 | 12,93 | 952,30 | 1122 |
| 75-79 | 0,015 | 0,007 | 0,016 | 0,003 | 0,003 | 4,73 | 643,16 | 824 |
| 80-84 | 0,019 | 0,017 | 0,033 | 0,008 | 0,006 | 7,31 | 417,46 | 524 |
| >85 | 0,016 | 0,013 | 0,033 | 0,004 | 0,008 | 2,89 | 220,96 | 310 |

|  | **Female** |  | 95% Confidence Interval | |  |  |  |  |
| --- | --- | --- | --- | --- | --- | --- | --- | --- |
| Age | Prob value | Incidence | Upper | Lower | SE | Alpha | Beta | PY |
| 55-59 | 0,004 | 0,003 | 0,007 | 0,001 | 0,001 | 5,51 | 1664,29 | 2128 |
| 60-64 | 0,005 | 0,004 | 0,007 | 0,001 | 0,002 | 5,39 | 1533,23 | 1980 |
| 65-69 | 0,003 | 0,004 | 0,008 | 0,002 | 0,002 | 5,51 | 1406,40 | 1787 |
| 70-74 | 0,003 | 0,005 | 0,010 | 0,002 | 0,002 | 6,44 | 1281,95 | 1587 |
| 75-79 | 0,004 | 0,005 | 0,011 | 0,002 | 0,002 | 5,54 | 1039,56 | 1309 |
| 80-84 | 0,004 | 0,004 | 0,011 | 0,001 | 0,003 | 2,94 | 681,10 | 930 |
| >85 | 0,008 | 0,008 | 0,019 | 0,003 | 0,004 | 3,80 | 459,84 | 612 |

| **Weighted Incidence** | |  |  |
| --- | --- | --- | --- |
| Age | Prob | deterministic | PYTotal |
| 55-59 | 0,005 | 0,004 | 4132 |
| 60-64 | 0,007 | 0,006 | 3705 |
| 65-69 | 0,008 | 0,006 | 3233 |
| 70-74 | 0,008 | 0,008 | 2709 |
| 75-79 | 0,003 | 0,006 | 2133 |
| 80-84 | 0,013 | 0,009 | 1454 |
| >85 | 0,011 | 0,010 | 922 |

| **Unisex weighted incidence per 10 years used in the model** | | |
| --- | --- | --- |
| Age | Probabilistic | Deterministic |
| 55-64 | 0,003 | 0,005 |
| 65-74 | 0,009 | 0,007 |
| 75-84 | 0,007 | 0,008 |
| >85 | 0,007 | 0,010 |

**Calculation of Relative Risk Reduction**

**Clopidogrel**

Source: Wong et al 2011^2^

Relative risk reduction in CV events by ASA 0.78

Relative Risk Reduction by Clopidogrel vs Aspirin 0.790

Relative Risk Reduction by Clopidogrel vs no treatment 1/((1/0.78)/0.79)

**= 0.616**

**References**

1. Meijer WT, Cost B, Bernsen RM, Hoes AW. Incidence and management of intermittent claudication in primary care in The Netherlands. Scandinavian journal of primary health care 2002;**20**(1):33-4.

2. Wong PF, Chong LY, Mikhailidis DP, Robless P, Stansby G. Antiplatelet agents for intermittent claudication. Cochrane database of systematic reviews 2011(11):CD001272.
